# A Novel Class of tRNA-Derived Small Non-Coding RNAs Respond to Myocardial Hypertrophy and Contribute to Intergenerational Inheritance

**DOI:** 10.3390/biom8030054

**Published:** 2018-07-16

**Authors:** Linyuan Shen, Mailin Gan, Zhengdong Tan, Dongmei Jiang, Yanzhi Jiang, Mingzhou Li, Jinyong Wang, Xuewei Li, Shunhua Zhang, Li Zhu

**Affiliations:** 1 College of Animal Science and Technology, Sichuan Agricultural University, Chengdu 611130, China; shenlinyuan0815@163.com (L.S.); 18299095425@139.com (M.G.); tzdabc123456@163.com (Z.T.); jiangdm2012@yahoo.com.cn (D.J.); mingzhou.li@163.com (M.L.); lixuewei9125@126.com (X.L.); 2 College of Life and Science, Sichuan Agricultural University, Chengdu 611130, China; jiangyz04@163.com; 3 Chongqing Academy of Animal Sciences, Chongqing 402460, China; kingyou@vip.sina.com

**Keywords:** tRNA fragments, myocardial hypertrophy, intergenerational inheritance

## Abstract

tRNA-derived fragments (tRFs) are a new class of non-coding RNA that play an important role in regulating cellular RNA processing and protein translation. However, there is currently no study reporting the influence of tRFs on myocardial hypertrophy. In this study, we used an isoproterenol (ISO)-induced myocardial hypertrophy rat model. Small RNA (<40 nts) transcriptome sequencing was used to select differentially expressed tRFs. We also compared the tRFs expression pattern in F0 sperm and the hearts of F1 offspring between the myocardial hypertrophy group (Hyp) and the control group (Con). Isoproterenol successfully induced a typical cardiac hypertrophy model in our study. Small RNA-seq revealed that tRFs were extremely enriched (84%) in the Hyp heart. Overexpression of tRFs1 and tRFs2 both enlarged the surface area of cardiac cells and increased expression of hypertrophic markers (*ANF*, *BNP*, and *β-MHC*). Luciferase reporter assay identified that tRFs1 directly target 3′UTR of Timp3. tRFs1, tRFs2, tRFs3, and tRFs4 were also highly expressed in Hyp F0 sperm and in Hyp F1 offspring hearts, but there was no differential expression of tRFs7, tRFs9, and tRFs10. Compared to Con F1 offspring, Hyp F1 offspring had elevated expression levels of *β-MHC* and *ANP* genes, and they had increased fibrosis and apoptosis in their hearts. These results demonstrated that tRFs are involved in regulating the response of myocardial hypertrophy. Besides, tRFs might serve as novel epigenetic factors that contribute to the intergenerational inheritance of cardiac hypertrophy.

## 1. Introduction

Cardiac hypertrophy leading to heart failure is one of the major causes of morbidity and mortality worldwide, and it is a common name used for cardiovascular diseases such as cardiomyopathies [1]. To date, despite significant progress in the treatment of heart failure, the underlying molecular mechanisms of cardiac hypertrophy are still poorly understood. In recent decades, due to next-generation sequencing (NGS) techniques, non-coding RNAs (ncRNAs), such as microRNAs (miRNAs) [2], long non-coding RNAs (lncRNAs) [3], and circular RNA (circRNAs) [4], have been suggested to play pivotal regulatory roles in cardiac development and in the progression of cardiac disease [5]. This supports the indispensable function of ncRNAs in the development of cardiac hypertrophy.

Interestingly, a new class of small RNAs distinct from canonical ncRNAs has been recently discovered. They are produced by stress-released ribonuclease that cleaves mature tRNA into fragments. Therefore, these tRNA-derived fragments were named tRFs [6]. Subsequent studies have shown that various stress conditions, such as chronic hepatitis, can specifically induce tRNA cleavage [7]. Additionally, tRFs in sperm could function as a paternal epigenetic factor, and they could mediate the intergenerational inheritance of paternal disease [8]. Besides, other studies proved that tRFs could serve as small interfering RNA that regulated protein translation and diverse biological processes [9]. However, there is currently no study that has investigated the involvement of tRNA-derived ncRNAs in myocardial hypertrophy.

The main objective of this study was to elucidate the function of tRFs in the development of myocardial hypertrophy and to determine a possible association with the intergenerational inheritance of this disease.

## 2. Results

### 2.1. Isoproterenol Induces Cardiac Hypertrophy and Influences Small RNA Distribution in SD Rats

**Figure 1:**
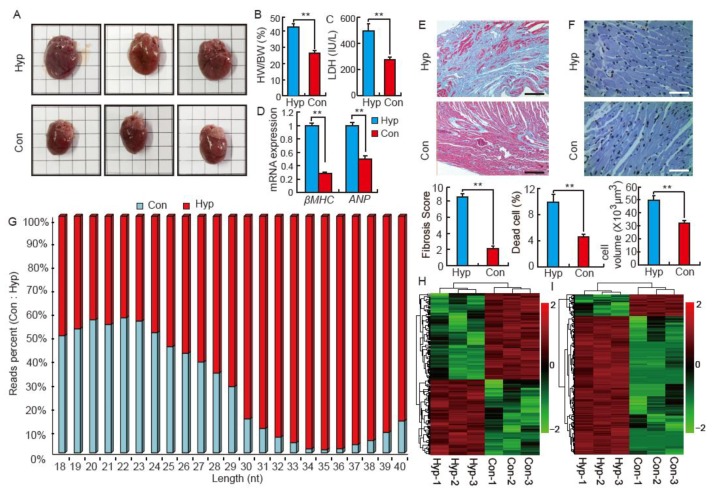
Isoproterenol (ISO) induces cardiac hypertrophy and influences small RNA distribution in SD rats. (**A**) Images of whole hearts from control (Con) and isoproterenol-induced (Hyp) Sprague Dawley (SD) rats, *n* = 8; (**B**) The ratio of the heart weight to body weight (HW/BW) in Con and Hyp groups, *n* = 8. Data are means ± SD. ** *p* < 0.01; (**C**) The activity of serum lactate dehydrogenase (LDH) in Con and Hyp groups, *n* = 8; (**D**) Relative expression of mRNA of *β-MHC* and *ANP*. The qRT-PCR analysis was performed in triplicate with three independent samples; (**E**) Cardiac fibrosis evaluated by Masson trichrome staining. Scale bar = 200 μm, *n* = 8; (**F**) Histological analysis of heart tissue in Hyp and Con groups by Terminal deoxynucleotidyl transferase dUTP nick end labeling (TUNEL) staining. Scale bar = 50 μm, *n* = 8; (**G**) Reads distribution of small RNA (18–40 nts) in the Con and Hyp groups, *n* = 3; (**H**) Hierarchical clustering analysis for differentially expressed miRNA in Con and Hyp groups, *n* = 3; (**I**) Hierarchical clustering analysis for differentially expressed tRFs in Con and Hyp groups, *n* = 3.

Isoproterenol (ISO)-induced rats had a significant (*p* < 0.01) increase in heart volume and weight (Figure 1A,B). ISO-induced rats also demonstrated a significant increase (*p* < 0.01) in the activity of serum lactate dehydrogenase (LDH, Figure 1C). The mRNA levels of hypertrophic markers, *β-MHC* and *ANP*, were significantly (*p* < 0.01) elevated in ISO hearts (Figure 1D). Moreover, the microscopic analysis demonstrated that ISO increased rat cardiac fibrosis, cell apoptosis, and myocyte area (Figure 1E,F). These results indicate that ISO induced a model of typical cardiac hypertrophy in our study. To identify the tRFs produced in response to the stress of cardiac hypertrophy, small RNA (<40 nts) transcriptome sequencing was conducted. We found no distinct difference in miRNA distribution (20–24 nts) between Hyp and Con hearts (Figure 1G). However, tRFs (32–38 nts) were extremely enriched in the Hyp group (Figure 1G). This is consistent with the distribution of differentially expressed miRNAs and tRFs in Con and Hyp hearts, respectively (Figure 1H,I).

### 2.2. tRFs Promote Cardiac Hypertrophy in Cardiomyocytes

**Figure 2:**
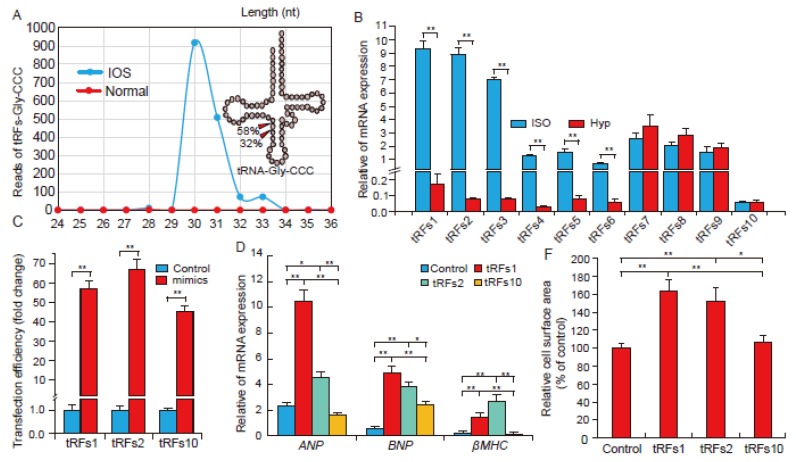

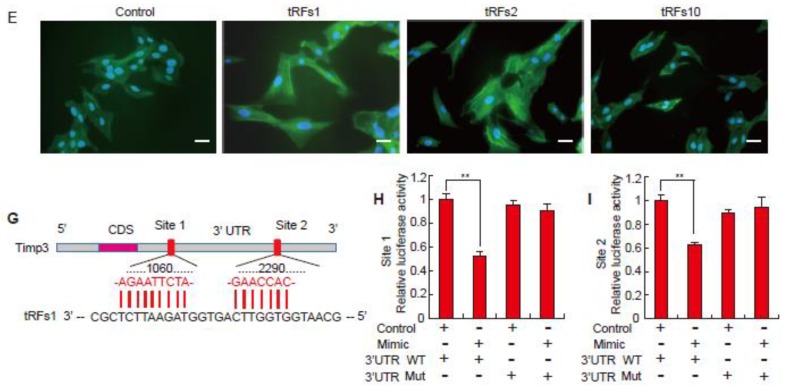
tRFs promote cardiac hypertrophy in cultured cardiomyocytes. (**A**) The distribution of different lengths of tRFs derived from tRNA-Gly-CCC; (**B**) Verification of the expression of tRFs by qRT-PCR, *n* = 3; (**C**) Transfection efficiency of tRFs mimics in the H9c2 cells, *n* = 3; (**D**) Relative mRNA expression of *ANP*, *BNP,* and *β-MHC* in treated H9c2 cells, *n* = 3; (**E**) Immunofluorescence evaluation of cardiac hypertrophy in cells treated with tRFs mimics. Bar indicates 50 μm, *n* = 3; (**F**) The relative cell surface area in treated H9c2 cells, *n* = 3; (**G**) Sequence alignment of tRFs1 with 3′-UTR of Timp3; (**H**) The repressive effect of tRFs1 on the activity of site1 of Timp3 3′UTR measured by luciferase assay, *n* = 3. (**I**) The repressive effect of tRFs1 on the activity of site2 of Timp3 3′UTR measured by luciferase assay, *n* = 3. Data are means ± SD. * *p* < 0.05, ** *p* < 0.01.

Based on the cleavage pattern of tRNA-Gly-CCC (Figure 2A), we found that tRNA-derived tRFs were not random products of tRNA fragments but the results of precise cleavage modulations in response to myocardial hypertrophy. To further validate RNA-seq results, the expression patterns of ten selected tRFs were validated by qRT-PCR (Table 1). The results indicate that the two methods were highly consistent (Figure 2B). To determine whether the top differentially expressed tRFs, tRFs1 and tRFs2, could accelerate myocardial hypertrophy, tRF mimics were overexpressed in H9c2 cells (Figure 2C). As shown in Figure 2D, the expression levels of hypertrophic markers such as *ANF*, *BNP*, and *β-MHC* were all elevated in tRFs1 and tRFs2 over-expression cells, but there was no effect on cells treated with tRFs10 mimics. Immunostaining with α-actinin antibody revealed significantly larger cell surface area in the tRFs1 and tRFs2 treated group compared to that in the control and tRFs10 treated group (Figure 2E,F). Besides, we found that Timp3, cardiac hypertrophy regulatory factor, had two potential target sites of tRFs1 (Figure 2G). According to luciferase reporter assay (Figure 2H,I), we confirmed that tRF1 inhibited Timp3 expression by directly targeting its 3′UTR.

### 2.3. The Function of tRNA-Derived Fragments on the Intergenerational Inheritance of Cardiac Hypertrophy

**Figure 3:**
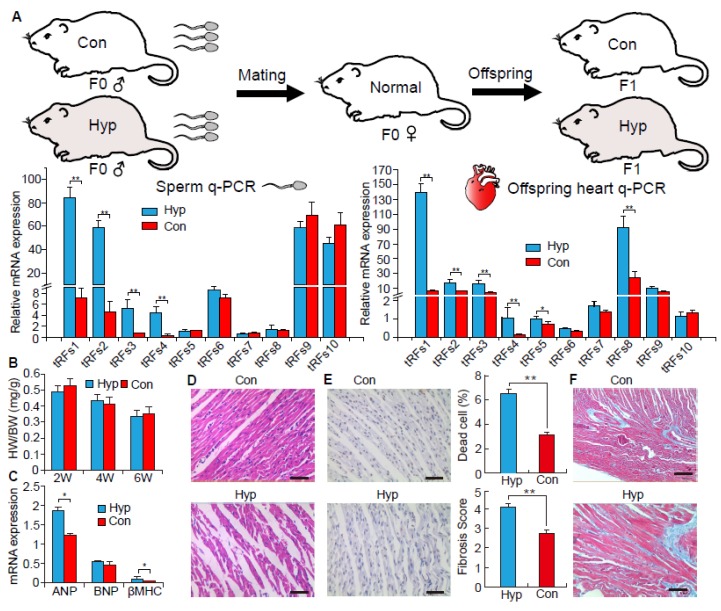
The function of tRFs on the intergenerational inheritance of cardiac hypertrophy. (**A**) The expression levels of tRFs in F0 sperm and F1 offspring heart, *n* = 8; (**B**) The ratio of the heart weight to body weight (HW/BW) in F1 offspring Con and Hyp groups, *n* = 8; (**C**) Relative mRNA expression levels of *ANP*, *BNP*, and *β-MHC* in F1 offspring heart, *n* = 8; (**D**) Histological analysis of the F1 offspring heart tissue by HE staining. Scale bar = 50 μm, *n* = 8; (**E**,**F**) Cell apoptosis and cardiac fibrosis analysis of F1 offspring heart using TUNEL staining and Masson staining, respectively. Scale bar = 50 μm, *n* = 8. Data are means ± SD. * *p* < 0.05, ** *p* < 0.01.

To further investigate whether sperm tRFs contribute to an intergenerational inheritance of myocardial hypertrophy, we used healthy F0 female rats and mated them with Hyp and Con F0 males to get Hyp and Con F1 rats, respectively. As shown in Figure 3A, most of the differentially expressed tRFs in the F0 heart were also highly expressed in the F0 sperm. This distinction was consistently inherited in the F1 offspring’s heart (Figure 3A). However, we found no differences in HW/BW between Hyp and Con F1 offspring (Figure 3B). Interestingly, there was increased expression of hypertrophic marker genes (*β-MHC* and *ANP*) in ISO F1 offspring as compared to Con F1 offspring (Figure 3C). HE staining suggests that ISO F1 offspring had an increase in the extent of heart muscle fiber breakage (Figure 3D). Terminal deoxynucleotidyl transferase dUTP nick end labeling (TUNEL) staining indicated that ISO F1 offspring exhibited a high proportion of apoptotic myocardial cells (Figure 3E). Masson trichrome staining suggested that ISO F1 offspring had significantly elevated fibrosis in their hearts (Figure 3F).

## 3. Discussion

tRFs are a novel class of non-coding RNA that are reported to participate in the regulation of RNA processing and protein translation [10,11]. They respond to many stress conditions, such as oxidative stress, ischemia, and chronic hepatitis [7,12,13]. In the current study, we are the first to report that tRFs also respond to the stress of cardiac hypertrophy. We found a robust increase in tRF expression in cardiac hypertrophy induced by ISO in rats, with about 84% of total tRFs highly expressed in the ISO group (Figure 1I). These findings are consistent with previous studies, which found that tRFs were highly enriched when exposed to stress conditions [7,13]. We also found that tRFs1 derived from tRNA-Gly-CCC was the highest expressed tRFs in the ISO heart, and its fold change was more than 868 (Table 1). As shown in Figure 2A, the primary cleavage site of tRFs1 was at 30 nucleotides (nt) (58%) and 31 nt (32%). This suggests that tRFs are not random products, but they have precise cleavage sites in response to the stress of cardiac hypertrophy. Moreover, these results also indicate that tRFs may play a distinctive role in response to stress and are not just RNA noise. In fact, we have demonstrated that tRFs1 and tRFs2 may enlarge the cell surface and increase the expression of hypertrophic genes in cardiomyocytes. In vivo animal studies have shown that knocking out Timp3 in mice would result in severe cardiac fibrosis and hypertrophy [14]. Here, we confirmed that tRFs1 could directly target 3′UTR of Timp3, which suggested that tRFs have similar functions with miRNAs in epigentic regulation. This finding was consistent with the previous study about tRFs function on gene regulation [15].

Sperm can be used as a carrier to introduce genetic material, and it possesses various types of genetic materials that can transmit information to offspring. For example, Rodgers et al. (2015) found that zygote microinjection of 9 specific sperm miRNAs (previously identified in paternal stress mouse) could induce a stress dysregulation phenotype in offspring [16]. Chen et al. (2015) injected sperm tRFs from high-fat diet fed male mice into normal zygotes and generated metabolic disorders in the F1 offspring [8]. Interestingly, in this study, we found that differentially expressed tRFs in sperm from cardiac hypertrophy rats had similar expression patterns in offspring. Although we did not observe a change in HW/BW between normal and cardiac hypertrophy offspring, the expression of hypertrophic marker genes (*β-MHC* and *ANP*) were significantly increased in Hyp F1 offspring (Figure 2C). Moreover, we found that Hyp F1 offspring had severe heart muscle fiber breakage and high levels of fibrosis according to histology analysis. Therefore, these results indicate that Hyp F1 offspring already possess some of the symptoms of cardiac hypertrophy. Although these preliminary results prove expression patterns of tRFs are closely related to offspring phenotypes, the conjecture of tRFs have the function on the intergenerational inheritance of cardiac hypertrophy is short of direct evidence. Furthermore, isoprenaline has side effects on the spermatogenesis [17]. Therefore, zygote microinjection of some tRFs to directly verify the phenotype of cardiac hypertrophy in offspring is needed in the future.

An important limitation of our study is lack of an experiment of zygote microinjection of some tRFs to verify the phenotype of cardiac hypertrophy in offsprings. Although these preliminary results prove that the expression patterns of tRFs are closely related to offspring phenotype, further studies including more genetic mechanism are required. Besides, the study design should add another control group (the presence of contaminant full length tRNA) to subtract the background from tRFs expression. Several identified tRFs are not uniquely related to tRNA sequences, which implies that there is need to prove these tRFs are only cleaved from tRNAs before to explore its function.

## 4. Materials and Methods

Following the Ethics Committee approval, under permit No. DKY-B20131403 (Ministry of Science and Technology, China, revised in June 2004). Six week-old male SD rats were randomly divided into two groups: Control (Con: saline administration through subcutaneous, *n* = 8) and Hypertrophy (Hyp: isoproterenol administration through subcutaneous, 5 mg/kg/day, *n* = 8). Rats had free access to food and water during the entire experimental period (14 days). Subsequently, the two groups were mated with healthy female SD rats to produce Con and Hyp F1 offspring. The offspring were maintained until 6 weeks of age at the same environment. F0 rats were sacrificed at 8 weeks, F1 rats were sacrificed at 2, 4, and 6 weeks. Following sacrifice, heart tissues were carefully isolated and used for the assessment of morphometric, biochemical, and gene expression analysis. Hearts were fixed in 4% paraformaldehyde for further histological analysis. HE staining was performed to measure the area and the breakage of muscle fiber. Masson trichrome staining (Sigma-Aldrich, St. Louis, MO, USA) was performed to evaluate cardiac fibrosis. TUNEL staining was used to measure apoptosis and to evaluate the cell surface area [18]. The concentrations of serum LDH (No. A020-1) were determined using commercial kits (Nanjing Jiancheng Institute of Bioengineering, Nanjing, China), following the manufacturer’s instructions. Small RNAs of 15–40 nts were excised from total RNA. They were used to construct a small RNA-sequence library and were sequenced on an Illumina Hi-Seq 2000 apparatus. The flow of small RNA-seq data processing and analysis was performed according to a previous study [8]. Small RNA-sequencing data was deposited on GEO (GSE106511).

Rat H9c2 cells (ATCC) were cultured in DMEM supplemented with 10% FBS (Invitrogen, Carlsbad, CA, USA). tRFs1, tRFs2, and tRFs10 mimics were synthesized by Ribobio (Ribobio, Guangzhou, China). Cell transfection was performed using the Lipofectamine 2000 reagent (Invitrogen, Carlsbad, CA, USA), according to the manufacturer’s protocol. Immunofluorescence was performed on H9c2 cells using anti-cardiac α-myosin heavy chain antibody (Abcam, Cambridge, UK). The relative cell area was analyzed by ImageJ 1.51 software (National Health Institutes, Bethesda, MD, USA). Luciferase reporter assay was performed according to our previous study [19]. Briefly, wild-type and mutant Timp3 3′-UTR were inserted into psiCHECK™-2 vector (Promega, Madison, WI, USA) between XhoI and NotI restriction sites respectively. For the luciferase reporter analysis, HeLa cells were respectively transfected with recombinant psiCHECK™-2 vector containing wild-type or mutant Timp3 3′-UTR, and tRFs1 mimic. At 24 h after transfection, luciferase activities were measured using the Dual-GloLuciferase Assay System (Promega, Madison, WI, USA), following the manufacturer’s instructions. tRFs quantification was performed using custom-designed TaqMan MicroRNA Assays, according to manufacturer’s recommended protocols. Reverse transcription was performed using the TaqMan MicroRNA reverse transcription kit and RT-PCR using TaqMan Universal PCR Master Mix (Applied Biosystems, Foster City, CA, USA). mRNA quantification was performed using the SYBR Premix Ex Taq kit (TaKaRa, Tokyo, Japan) on the CFX96 system (Bio-Rad, Hercules, CA, USA). Endogenous U6 and GAPDH were used as internal controls. Relative expression levels of tRFs and mRNA were calculated using the 2^−ΔΔCt^ method. Data are presented as the mean ± standard deviation (SD). Data were analyzed using one-way analysis of variance (ANOVA) to test the homogeneity of variances followed by Student’s *t*-test analyses in SPSS (21.0 version, IBM, Armonk, NY, USA). Statistical significance was set at *p* < 0.05.

## 5. Conclusions

In conclusion, our study is the first to investigate the function of tRFs in myocardial hypertrophy. We found that tRFs were extremely enriched in hearts with cardiac hypertrophy, and they may enlarge myocardial cell surface area and the expression of hypertrophic genes. Interestingly, sperm tRFs might serve as an epigenetic factor that transmits the symptoms of cardiac hypertrophy to offspring. Thus, this study may have implications for cardiac hypertrophy pathogenesis and novel therapeutic strategies.

## Figures and Tables

**Table 1:** List of some interesting tRNA fragments (tRFs).

Name	Sequence	Length	tRNA Mapped	Fold Change
tRFs1	GCAATGGTGGTTCAGTGGTAGAATTCTCGC	30	tRNA-Gly-GCC	867.53
tRFs2	TCCCATATGGTCTAGCGGTTAGGATTCCTGGTTT	34	tRNA-Glu-TTC	415.64
tRFs3	TCCATGGTGGTCTAGTGGTTAGGATTCGGC	30	tRNA-Glu-CTC	352.76
tRFs4	GGTTCCATGGTGTAATGGTTAGCACTCTGGACTC	34	tRNA-Gln-CTG	137.83
tRFs5	GCACTGGTGGTTCAGTGGTAGAATTCTCGC	30	tRNA-Gly-CCC	124.92
tRFs6	GTTTCCGTAGTGTAGTGGTTATCACGTTCGCCTC	34	tRNA-Val-CAC	96.76
tRFs7	ATTAGGGTGGCAGAGCCAGGTAATT	25	tRNA-Leu-TAA	0.94
tRFs8	GTAGTCGTGGCCGAGTGGTTAAG	23	tRNA-Ser-AGA	0.98
tRFs9	TAGGATAGGGTGTATTGGTAGCAC	24	tRNA-Gln-TTG	1.04
tRFs10	TTGGGGTGCGAGAGGTCCCGGGTT	24	tRNA-Pro-AGG	1.07
